# Effects of Pile Driving on the Residency and Movement of Tagged Reef Fish

**DOI:** 10.1371/journal.pone.0163638

**Published:** 2016-11-23

**Authors:** Joseph D. Iafrate, Stephanie L. Watwood, Eric A. Reyier, Douglas M. Scheidt, Georges A. Dossot, Steven E. Crocker

**Affiliations:** 1 Naval Undersea Warfare Center Division, Newport, Rhode Island, United States of America; 2 Kennedy Space Center Ecological Program and Integrated Mission Support Services, Kennedy Space Center, Florida, United States of America; Virginia Commonwealth University, UNITED STATES

## Abstract

The potential effects of pile driving on fish populations and commercial fisheries have received significant attention given the prevalence of pile driving occurring in coastal habitats throughout the world. Behavioral impacts of sound generated from these activities on fish typically have a greater area of influence than physical injury, and may therefore adversely affect a greater portion of the local population. This study used acoustic telemetry to assess the movement, residency, and survival of 15 sheepshead (*Archosargus probatocephalus*) and 10 grey snapper (*Lutjanus griseus*) in Port Canaveral, Florida, USA, in response to 35 days of pile driving at a wharf complex. No obvious signs of mortality or injury to tagged fish were evident from the data. Received sound pressure levels from pile strikes on the interior of the wharf, where reef fish primarily occur, were on average 152–157 dB re 1 μPa (peak). No significant decrease in sheepshead daytime residency was observed during pile driving within the central portion of the wharf and area of highest sound exposure, and no major indicators of displacement from the exposure wharf with the onset of pile driving were observed. There was evidence of potential displacement from the exposure wharf that coincided with the start of pile driving observed for 2 out of 4 grey snapper, along with a decrease in daytime residency for a subset of this species with high site fidelity prior to the event. Results indicate that snapper may be more likely to depart an area of pile driving disturbance more readily than sheepshead, but were less at risk for behavioral impact given the lower site fidelity of this species.

## Introduction

The increasingly common occurrence of pile driving is related to intensified urban development in coastal areas, as well as construction of renewable energy facilities in offshore waters. Driving a pile into the sediment with an impact hammer introduces high intensity impulsive sound waves into the water column that result in a rapid rise in pressure. It has been shown that high intensity impulsive sounds, such as from pile driving, can potentially cause injury in fish at high received levels [1–3]. For example, onset of tissue damage in Chinook salmon for average single strike sound exposure in a controlled laboratory simulation was observed at 177 and 180 dB re 1 μPa^2^·s for 1920 and 960 strikes, respectively [3]. The severity of exposure and potential for injury may also be greater in shallow water than deep water environments due to decreased hydrostatic pressure [3].

While an impulsive source and the resulting pressure waves could lead to injury to those individuals at close range, behavioral changes can occur at greater distances from the source and therefore may affect a larger portion of a population, by causing movement of fish away from feeding or breeding grounds or changes in migratory behavior [4–6]. There can be negative population-level repercussions if fish displace to nearby areas that are already at carrying capacity or offer sub-optimal conditions. Additionally, consequences could be more severe if pile driving occurs within critical habitat of endangered species or if migratory corridors (e.g., river mouths) are affected.

Although the value of 150 dB re 1 μPa has commonly been used as the threshold for behavioral response in fish for U.S. regulatory analyses [7], the origin of this value is unknown [4], and there is a lack of applicable behavioral studies that examine the effects of high intensity sounds on fish in the wild [4–6, 8]. Notable studies on behavioral response to pile driving thus far either occurred in enclosed environments where behavior cannot be confidently extrapolated to the normal behavior of wild animals, or used playbacks of pile driving sounds that do not account for the additional energy from ground-borne vibrations associated with pile driving [9]. Results for these studies include impaired startle response for seabass (*Dicentrarchus labrax*) [10], significant freezing response for tagged and penned Atlantic cod (*Gadus morhua*) and increase in swimming speed for sole (*Solea solea*) to playback of pile driving sounds at received sound pressure levels of 140–161 dB re 1 μPa and 144–156 dB re 1 μPa, respectively [11], and no significant changes in behavior for caged coho salmon (*Oncorhynchus kisutch*) [12], or brown trout (*Salmo trutta*) [13]. Hawkins et al. [14] used sonar to find that fish schools of sprat (*Sprattus sprattus*) and Atlantic mackerel (*Scomber scombrus*) subject to playback of simulated pile driving responded on 50% of presentations at received levels of 163.2 and 163.3 dB re 1 μPa peak-to-peak and 135.0 and 142.0 dB re 1 μPa^2^·s single strike sound exposure level, respectively. Still, the potential displacement of fish in the wild from a high-intensity, sustained sound source or potential habituation to the sound over time in the natural environment have not been examined. Given the lack of clear behavioral thresholds, Popper et al. [4] recently outlined the relative risk for behavioral impacts from pile driving based on distance from the source in what is considered interim sound exposure guidelines for fishes.

This study documents the survival, residency and movement of free-ranging perciform reef fishes during pile driving. Sheepshead (*Archosargus probatocephalus*) and grey snapper (*Lutjanus griseus*) were chosen as target species due to their abundance, their membership in diverse reef fish families (Sparidae and Lutjanidae), and high site fidelity to hardbottom habitats [15,16]. Sheepshead are known to be strongly associated with structures such as pilings, jetties, and oyster reefs [17], and are considered highly dependent on sessile invertebrates such as barnacles as a food source [18]. Grey snapper also seek structure particularly when aggregating in resting schools during daylight hours, but more readily move between a variety of habitats [19]. Species from both families inhabit marine and estuarine habitats in tropical and subtropical areas throughout the world. The hearing range of both grey snapper and sheepshead would be considered similar to most fish species from below 100 Hz to several hundred Hertz [4]. The main goals of this study were to: (1) determine sound levels that free-ranging fish present at the construction wharf were exposed to during pile driving, (2) examine short-term survival of fish within the region of influence during pile driving, (3) monitor for displacement of animals from the immediate area, and (4) compare residency of fish before, during, and after exposure to pile driving.

## Materials and Methods

### Ethics Statement

Fish collection and handling was performed in accordance with a State of Florida Special Activity License (permit SAL-09-0512A-SR) and the study was approved by Kennedy Space Center Institutional Animal Care and Use Committee (permit GRD-06-049).

### Study Area

**Fig 1:**
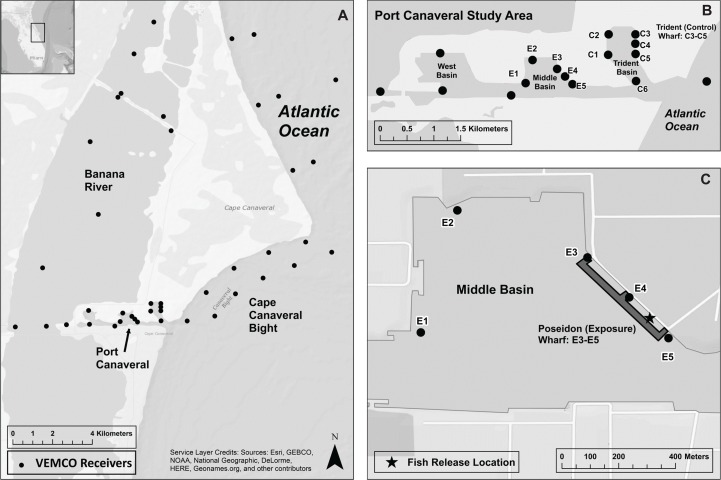
Port Canaveral study area. A) Regional study area including telemetry receivers in the Florida Atlantic Coast Telemetry Array. B) Port Canaveral Study Area. C) Middle Basin and Poseidon Wharf where pile driving occurred (darker shaded region represents marine waters). *Original figure adapted for reuse with permission of Springer from*: *The Effects of Noise on Aquatic Life II*. *Volume 875 of the series Advances in Experimental Medicine and Biology pp 479–487*. *Residency of Reef Fish During Pile Driving Within a Shallow Pierside Environment*. *Joseph D*. *Iafrate*, *Stephanie L*. *Watwood*, *Eric A*. *Reyier*, *Matthew Gilchrest*, *Steven E*. *Crocker*. *© Springer Science+Business Media New York 2016*. *Figure was created using ArcGIS® software by Esri*. *ArcGIS® and ArcMap™ are the intellectual property of Esri and are used herein under license*. *Copyright © Esri*. *All rights reserved*.

Port Canaveral is an entirely manmade multi-use harbor that supports cruise ships, cargo and fishing vessels, and military activity. The Port is located in east-central Florida (28.5° N, Fig 1) and is comprised of a main navigation channel running east-west connecting three vessel turning basins, and dredged to a maximum depth of 14 m mean low water. Bottom substrates in the dredged Port Canaveral harbor are fine sediment sand or muddy sand. The West Basin contains several cruise ship terminals, while Poseidon and Trident Wharfs in Middle and Trident Basins, respectively, are managed by the Naval Ordnance Test Unit as a military facility, and therefore are closed to the public. The wharfs are massive structures of similar size at 366 m long by 40 m (Poseidon) and 50 m (Trident) wide supported underneath by multiple rows of large concrete piles providing refuge and forage for reef fish. Open spaces 40 m wide exist along the interior of the wharfs abutting the seawall. The Poseidon (exposure) Wharf was the location of pile driving for this study, while the Trident (control) Wharf served as an adjacent control site. The expansive wharfs and adjacent stone revetments within the Port create valuable hard-bottom habitat which support persistent populations of tropical reef fish species. The target species in particular have strong fidelity to the wharfs and interior open spaces, and were regularly visually observed in this habitat during daytime collection hours. While these fish are regularly exposed to anthropogenic noise from vessel activity within Port Canaveral, events that produce sound at the source levels typical of pile driving are not common. No pile driving occurred in the control basin concurrent with the pile driving along the exposure wharf.

### Pile Driving Event

The pile driving event was part of a fender replacement project at the exposure wharf which removed and replaced wooden piles with 104 polymetric fiberglass-reinforced concrete piles. The square piles were 41 centimeters on edge by 24 m in length and were placed in two main sections along the center and northern exterior face of the exposure wharf. Turbidity curtains were deployed around the pile extraction and placement operations. The approximate water depth along the outside of the wharf was 10 m. Piles were installed within a 35 day period in November and December 2011 during daylight hours only (0800–1700 EDT). On average, 5–7 piles were driven per day (maximum of 10), with an average of 200 strikes/pile. Piles were installed to within 10–15 feet of total depth with high-pressure jetting, and then a series of strikes with a PileMaster 36–5000 air impact hammer set the piles, with an approximate 6–7 minute install time and a blow rate of 30–45 blows per minute. This air impact hammer has an energy rating of 762–4572 meters per pound.

### Acoustic Recording

Sound pressure recordings in the exposure basin were made during four days of the event at distances of 10 to 370 m from the pile driving, using two calibrated Cetacean Research Technology C55 hydrophones (Cetacean Research Technology, Seattle, WA [mean sensitivity -165 decibels referenced to 1 volt per microPascal (dB re: 1 V/μPa)]), two calibrated High Tech, Inc. HTI-96-Min hydrophones (High Tech, Inc, Long Beach, MS [mean sensitivity -185 dB re: 1 V/μPa]), and a DT9837 4-channel dynamic signal acquisition module (Data Translation, Marlboro, MA). Hydrophones were calibrated at the Naval Undersea Warfare Center Underwater Sound Reference Division facility in Leesburg, FL. The lower sensitivity High Tech, Inc. hydrophones were used to reduce clipping of recorded data at close range to the source.

Pile driving source levels were measured at 10 m from impact pile driving for two different piles, with approximately 120 strikes for each pile. Sound levels during pile driving were recorded adjacent to telemetry receivers E3, E4, and E5 on the interior of the wharf (Fig 1) and in open water areas of the exposure basin to the west of the wharf. These values are dependent on the distance to the pile being driven while recordings were made, and therefore the received levels presented for individual receivers do not represent the full range of sound exposure at each location over the duration of the event, as recordings were not made for every pile driven over the 35 days. The range of distances between driven piles and exposure wharf telemetry receivers over the duration of pile driving were as follows: E3 = 50–206 m; E4 = 45-180m; E5 = 180-362m. Ambient sound recordings in open water areas of the exposure basin were collected when no piles were being driven.

A custom written algorithm to detect peak energy was used to locate the pile driving impacts in the recorded time series data in MATLAB (Mathworks, Natick, MA). Frequency dependent hydrophone response curves were applied, and recordings where the signal was clipped due to overloading of the recording system were excluded from analysis. Peak sound pressure level (SPL) and sound exposure level (SEL) were calculated for each impact on each of four (open water) or three (interior wharf) hydrophones located at different spanning the water column and then averaged for final results. Depths for recordings in open water were 2.1, 3.7, 5.2, and 6.7 m, while depths of hydrophones in the interior spaces of the wharf were 0.9, 2.4, and 4.0 m. Sound metrics presented in this study are defined as the following:

• *Peak SPL* is the maximum absolute value for the logarithmic measure of the instantaneous sound pressure for an individual pile strike. Sound pressure band levels (dB re 1 μPa) were computed in 1/3^rd^ octave bands ranging from center frequencies of approximately 50 to 5161 Hz [1]. The unit is dB re 1 μPa.

• *SEL* is equivalent to the time integral of the pressure squared for an impulse, and is calculated by summing the cumulative pressure squared over the duration for a single strike or single pile [1]. The unit is dB re 1 μPa^2^·s.

### Acoustic Telemetry

Vemco VR2W autonomous telemetry receivers (VEMCO Division, AMIRIX Systems Inc., Nova Scotia) were deployed throughout the port (Fig 1). These receivers record time of detection of uniquely coded acoustic transmitters. Receivers were bottom-mounted and secured via guy anchors with the exception of receiver C6, which was secured to a large moored stationary float in the entrance channel of the control basin. Range testing in both the exposure and control basins determined that greater than 50% of transmitter emissions were detected at distances up to 200 m in open water areas of the basin. However, stationary transmitters in the center of the exposure basin were not detected on receivers on the inner wharf, both during pile driving and with no pile driving, likely due to shadowing or interference from the concrete piles under the exterior portion of the wharf. Transmitters deployed on the outside face of the wharf were also not detected on inner wharf receivers. The maximum distance for detection on the interior of the wharf was 75m (>10% detects). Stationary transmitters were placed directly next to receivers during pile driving to independently confirm recording of data on acoustic receivers at close range to the source. Movement of a few fish that left the Port Canaveral study area were detected on VEMCO VR2W receivers maintained by the Florida Atlantic Coast Telemetry (FACT) Array, a regional-scale telemetry array maintained by several marine research organizations (Fig 1).

Fifteen sheepshead and 10 grey snapper were tagged in the exposure basin, and 12 sheepshead and 3 grey snapper were tagged in the control basin. All fish were collected either by gill net (31) or hook-line angling (9). Target fish had a minimum weight of 300 grams to ensure that the VEMCO V92L acoustic transmitters (length 29 millimeters; weight in air 4.7 grams) accounted for no more than the recommended 2% of in air body weight (Winter 1983). Battery life for all transmitters was an estimated 345 days. Fish were anesthetized in a solution of 75 milligrams tricaine methanosulfonate (MS-222; Western Chemical Inc.) per liter of sea water and placed ventral side up for surgical implantation of transmitters while gills were irrigated with fresh seawater. Incisions were closed with two absorbable sutures followed by application of tissue adhesive and triple antibiotic ointment, and fish were allowed to fully recover in aerated seawater prior to release. Standard length, fork length, weight, method of capture, and release condition were recorded for all fish. Fish were tagged nine to eleven days prior to the start of pile driving to ensure sufficient baseline data were obtained. No immediate mortality was observed due to handling or surgical implantation procedures.

### Behavioral Analyses

Raw detection data were filtered prior to conducting analyses to remove potential false-positive detections (single coded detections at an individual receiver within a 30-minute window). Two fish were caught and harvested by anglers, and were removed from data analyses subsequent to these catch dates. For fish released in the exposure basin, only 0.03% of sheepshead detections and 0.01% of snapper detections occurred on receivers E1 and E2, while detections on receivers C1 and C2 for sheepshead and snapper released in the control basin accounted for only 0.27% and less than 0.01% of detections, respectively. Therefore, movement and residency analyses focused only on wharf receivers at the exposure (E3, E4, E5) and control (C3, C4, C5) wharfs.

Residency index (RI) was calculated for each fish to represent the proportion of days an individual fish was detected on a receiver for a specific time period. RI was calculated for the daytime hours corresponding to the active pile driving schedule (0800–1700) at individual wharf receivers and the combination of three receivers located at the wharfs (E3-E5 and C3-C5). A non-parametric repeated measures one-way ANOVA (Friedman test) was used to test for significant differences in the median RI for sheepshead between the pre, during, and post-pile driving time periods. Significant differences were considered at an alpha level of 0.017 after Bonferroni correction for multiple comparisons. Time periods for this analysis were delineated as daytime periods for the following: pre-pile driving days 1–11 (1–11 November 2011); during-pile driving days 12–47 (12 November– 16 December 2011); and post-pile driving days 48–82 (17 December 2011–20 January 2012).

The number of residency periods per day (RP/D) was analyzed as an indicator of movement for those sheepshead present. RP/D was not examined for grey snapper due to the small sample present during pile driving. A residency period is defined as a continuous period of detections on a single receiver in which detections are less than 30 minutes apart. The number of residency periods within the basin may serve as an indicator of movement, as a residency period is recorded when an individual fish leaves the detection radius of a receiver, and either returns to the same receiver or moves to a different receiver after a period of time. Values for RP/D were only calculated for days sheepshead were detected during daylight hours. The pile driving period was also assessed in four equal time periods (quarters) for potential changes over time. Only sheepshead with data for all time quarters were included in comparisons. These equivalent periods were examined for potential habituation of fish over the duration of pile driving. The non-parametric Kruskal Wallis test was used for comparison between temporal groups for sheepshead for those individuals present at the exposure wharf.

## Results

### Sound Recording and Received Levels

**Fig 2:**
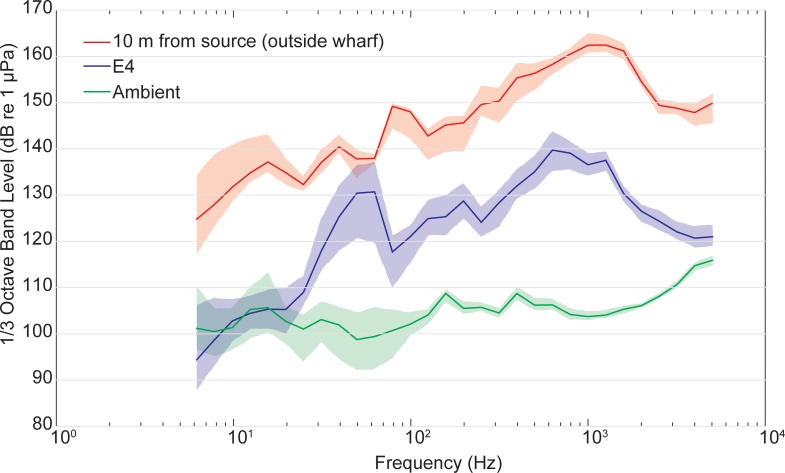
Median one-third octave band levels. Data are shown for pile strikes made 10 m from the source (orange), near receiver E4 on the interior of the wharf (purple), and during periods of no pile driving (ambient; green). Shaded regions represent 25^th^ and 75^th^ percentiles.

The measured mean peak source level determined from 240 strikes total from two piles was 175 ± 4.0 dB re 1 μPa at 10 m (Table 1). The peak energy levels from the pile driving strikes based on recordings of sound pressure were found to be within the 600–1200 Hz range (Fig 2). The ambient one-third octave band levels across the frequency range of interest related to fish hearing (100 Hz– 1000 Hz) are well below the one-third octave band levels for pile driving events at this location (Fig 2).

**Table 1:** Average peak received levels recorded at 10 m from pile driving and at locations internal and external to the exposure wharf.

Location (or closest receiver)	Water Depth (m)	Distance from pile driving (m)	Mean Peak SPL (± SD; dB re 1 μPa)	Mean Single Strike SEL(± SD; dB re 1 μPa^2^·s)
10 m	10.1	10	175 ± 4	149 ± 3
E3	4.3	179	152 ± <1	120 ± <1
E4	4.6	36	157 ± 2	139 ± <1
E5	4.3	194	153 ± 1	133 ± 5
Open water 1	10.9	41	165 ± 8	142 ± 7
Open water 2	10.9	78	162 ± 8	139 ± 4
Open water 3	13	371	144 ± <1	120 ± <1

Received levels as shown are considered representative for each location on select days of pile driving that recordings were made. Open water recordings were collected in the exposure basin to the west of the wharf with no obstructions to the source.

Based on measured recordings along the outer and interior wharf, it is likely that fish present within the interior of the exposure wharf were repeatedly exposed to levels in the range of 152 dB to 157 dB re 1 μPa peak SPL over the duration of pile driving, and on average 175 ± 4 dB re 1 μPa peak SPL on the outer fringes of the wharf closer to the pile being driven. Receiver E4 at the center of the exposure wharf was located in the closest proximity to active pile driving for the longest duration, followed by E3 and E5, respectively. During pile driving in the central portion of the outer wharf, the mean measured received level at receiver E4 was 157 ± 2.0 dB re 1 μPa peak SPL, as compared with 152 ± <1 dB and 153 ± 1.0 dB re 1 μPa peak SPL near receivers E5 and E3, respectively. Over time, these sound pressure levels translate to an estimated cumulative SEL of 162 dB re 1 μPa^2^·s per pile in the middle interior portion of the wharf, based on an average of 200 strikes/pile and an average single strike SEL of 139 dB re 1 μPa^2^·s. On average, received levels (mean peak SPL) on the interior were more than 8 dB re 1 μPa less than at a similar distance exterior to the wharf, although higher variability was observed for open water measurements (Table 1; Open water 1 vs. E4).

Received levels from pile driving activity were highly variable at each site and also between recording locations, particularly near receivers E3 and E5. This is likely due to changes in strike intensity resulting in fluctuating source levels in the relatively shallow waters and complex acoustic environment of the basin. Measured sound levels near pile driving activity with an unobstructed acoustic path showed direct broadband acoustic arrivals, while recordings at further distance in open water areas west of the wharf showed broadband arrivals with strong reflections. Overall, received levels in the inner wharf were reduced as compared with open water. Broadband transmission loss values calculated from the empirical data in open water areas were found to be highly variable, but generally were within the expected values between cylindrical and spherical spreading (Least squares fit: RL = SL– 18.64 log (r)). Spreading loss for areas on the interior wharf (receiver E4) was greater than for open water areas (RL = SL—23.10 log (r)).

### Fish Detection and Monitoring

**Fig 3:**
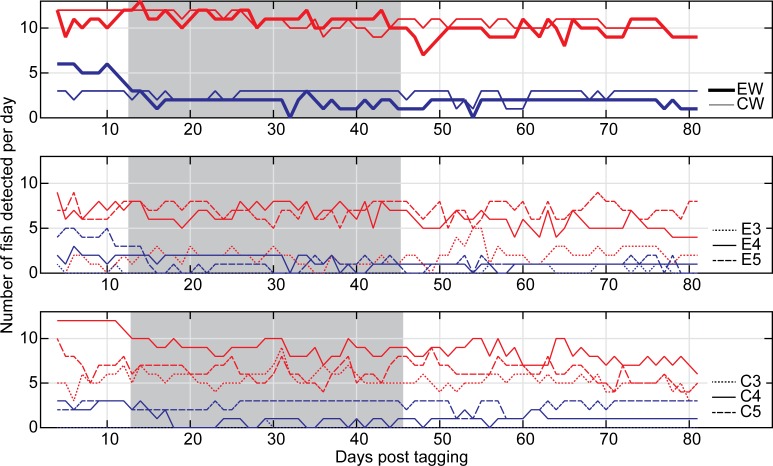
Number of tagged individual sheepshead and snapper detected per day at the exposure and control wharfs and corresponding individual receivers during the study period. Red lines represent sheepshead, blue lines represent snapper. Shading indicates the period of pile driving. A) Number of fish detected per day at the exposure wharf (EW) and the control wharf (CW). B) Number of fish detected per day at individual receivers on the exposure wharf. C) Number of fish detected per day at individual receivers on the control wharf.

In the exposure basin, the mean number of unique sheepshead detected per day on the exposure wharf remained fairly consistent at 10.1 before pile driving, with 9.9 and 9.5 individuals per day detected during and after pile driving, respectively. The number of sheepshead detected per day for each quarter of pile driving at the wharf in the exposure basin was also relatively stable, with overall values of 10.6, 9.4, 9.4, and 10.0 fish per day over time (Fig 3). During pile driving, the highest mean number of unique sheepshead detected per day was on receiver E5 (closest to the release site) followed by E4 located in the center of the wharf. The mean number of unique grey snapper detected per day on the exposure wharf decreased from 5.6 before pile driving to 1.9 during, followed by 1.8 after pile driving. The average number of snapper detected per day at the exposure wharf for each quarter of pile driving was 2.3, 2.0, 1.7, and 1.4 fish per day over time. As with sheepshead, the highest number of snapper detected per day for the duration of the study was on receiver E5 followed by E4 located in the center of the wharf.

The number of sheepshead detected daily over the entire control wharf (receivers C3-C5) decreased from 10.9 to 9.5 and 8.9 in the pre-, during-, and post-pile driving periods. For the duration of the study, the highest number of sheepshead detected per day was on receiver C4 located in the center of the wharf, followed by C5 to the south. Overall, fish in the control basin appeared to exhibit higher site fidelity. Of the 15 sheepshead tagged in the exposure basin, at most only 12 fish were ever detected on a single day, and only within the first three days of pile driving. In contrast, all 12 sheepshead tagged in the control basin were detected on multiple days in the first two weeks after tagging was completed. All three snapper released in the control basin were detected over the entire control wharf (receivers C3-C5) pre and during pile driving, while two fish were detected after pile driving. Only one fish was detected on C3, while all tagged snapper were detected on C4 and C5.

**Fig 4:**
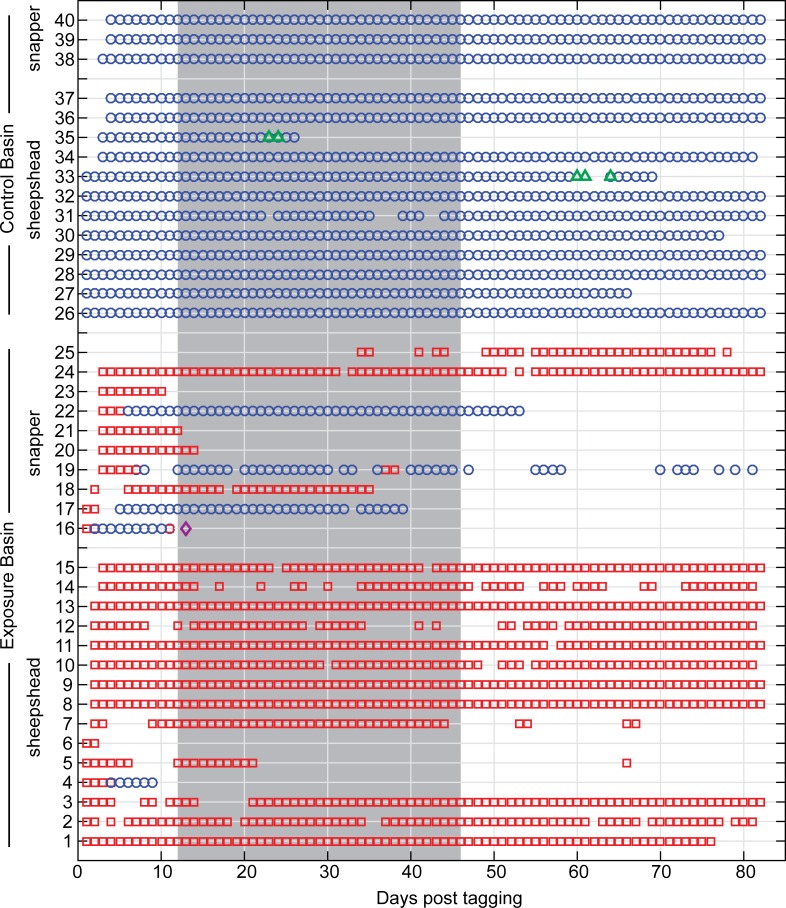
Acoustic detections of all 40 fish pre, during, and post pile driving. Detections shown for the exposure basin (red squares), the control basin (blue circles), Cape Canaveral Bight (green triangles), and Sebastian Inlet (purple diamond; 60 km south of Cape Canaveral). Each symbol represents a single day fish was present; shading indicates the period of pile driving.

Individual detection data were examined to determine timing of departure from the exposure basin (Fig 4). Two sheepshead left the exposure basin before pile driving began; sheepshead ID4 moved into the control basin while sheepshead ID6 was never detected again. The remaining sheepshead stayed in the exposure basin for the duration of pile driving, with the exception of sheepshead ID5 that left on day 10 of pile driving, and returned 21 days after pile driving ended.

Five snapper left the exposure basin prior to the start of pile driving; four moved into the control basin (snappers ID16, 17, 19, and 22), while snapper ID23 was never detected again. Snapper ID16 may have been subject to predation, given the brief time between detections at the study area and Sebastian Inlet (it would have moved ~60 km south in less than 2 days). However, this fish was also the largest snapper tagged at 325 mm SL. Of the four snapper present at the start of pile driving, snapper ID24 stayed in the exposure basin for the duration of pile driving, while snappers ID21, 20, and 18 left on day 1, 3, and 24 of pile driving, respectively. Finally, snapper ID25 did not have a first detection until 34 days post tagging (day 22 of pile driving), but appeared to remain in the exposure basin until after pile driving ended.

### Survivorship

Sheepshead released in the exposure and control basins averaged 191 and 156 days at liberty, respectively, compared to 130 and 276 days for grey snapper (Table 2). Additionally 87% of sheepshead released in the exposure basin were detected there after the final day of pile driving, as compared to 60% of grey snapper. Sixty-six percent of sheepshead released in the exposure basin were detected 3-months post tagging (45 days after pile driving ended) and 47% were detected 6-months post tagging (4.5 months after pile driving ended). Fifty percent of snapper released in the exposure basin were detected three-months post tagging and 30% were detected six-months post tagging. Seventy percent of sheepshead released in the control basin were detected 3-months post tagging and 40% were detected six-months post tagging. All three snapper released in the control basin were detected at least six months post-tagging. Eight fish were monitored for the full duration of tag battery life, with a maximum of 355 days for sheepshead and 353 days for snapper.

**Table 2:** Summary information for all tagged fish in the exposure and control basin.

	*Exposure Basin*		*Control Basin*	
	Sheepshead	Grey Snapper	Sheepshead	Grey Snapper
**Number Released**	15	10	12	3
**SL (mm; mean ±SD)**	232 ± 51	220 ± 45	244 ± 51	222 ± 4
**Days at Liberty (mean [SD])**	191 ± 145	130 ± 117	156 ± 109	276 ± 67
**Number of Detections (mean [SD])**	38,979 ± 77,769	11,336 ± 20,099	70,861 ± 63,478	56,557 ± 26,760
**Stations Visited (mean[SD])**	3 ± 0.8	4 ± 2.8	6 ± 1.8	4 ± 1.2

Days at Liberty equals the number of days between release and last detection.

### Residency and Movement

**Fig 5:**
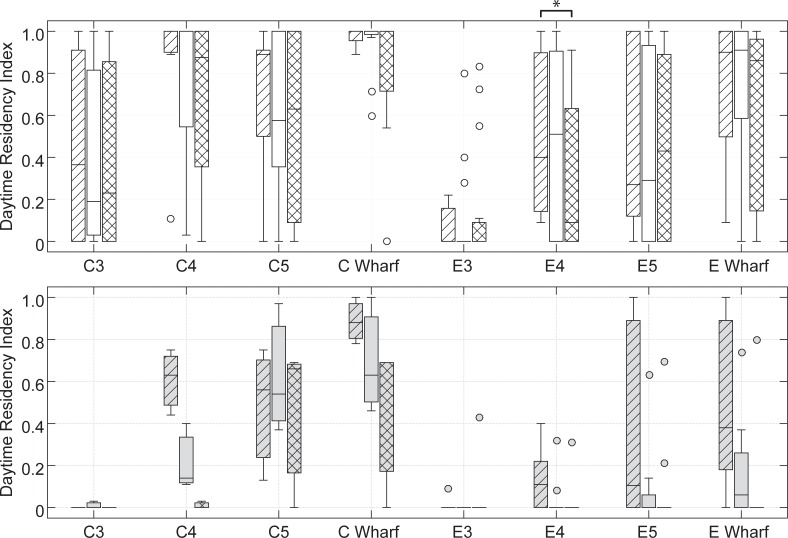
Daytime residency index (daytime hours only). Residency index is shown for sheepshead (top) and grey snapper (bottom) during the periods pre- (diagonal lines), during- (open), and post-pile driving (cross-hatched) for the three individual receivers at each wharf and the wharfs as a whole. Lines represent the median, boxes indicate the 25^th^ and 75^th^ interquartile range, whiskers extend to data within 1.5 times the interquartile range, and circles indicate outliers. Asterisk indicates significance at p<0.01.

Prior to pile driving, 11 of 15 sheepshead tagged at the exposure wharf were considered to have high site fidelity (daytime RI > 0.5) to the wharf, with seven of those fish also showing high site fidelity to the central portion of the wharf (E4). High site fidelity to the exposure wharf prior to pile driving was observed in only four out of 10 snapper, although none of these fish exhibited high site fidelity to the central portion of the wharf (E4). There were significant differences in daytime RI for sheepshead among time periods in the exposure wharf on receiver E4 (H = 8.44, df = 2, P = 0.02), while comparisons for the other receivers were non-significant (Fig 5). Only the pre-post pile driving reduction in RI for sheepshead at E4 was significant in post hoc analysis (z = -2.55, n = 15, P = <0.01). There were significant differences among time periods in the exposure wharf for grey snapper (H = 13.5, df = 2, P = <0.01), as the median RI decreased pre-post-pile driving on E5 (0.11 to zero), E4 (0.11 to zero), and the exposure wharf (0.38 to zero). Additionally, there was a notable decrease in daytime RI values in all four snapper with high site fidelity prior to pile driving, with three of these fish exhibiting daytime RI values of 0.0–0.03 during pile driving. However, none of the differences were significant in post-hoc analysis. Only one snapper was detected in the pre and post period at receiver E3.

At the control wharf, there were no significant differences in daytime RI among time periods for sheepshead at any of the individual or combined wharf receivers. There were also no differences in daytime RI for sheepshead across basins during pile driving. Analysis of grey snapper daytime RI in the control basin was not performed due to the small sample size (n = 3).

**Fig 6:**
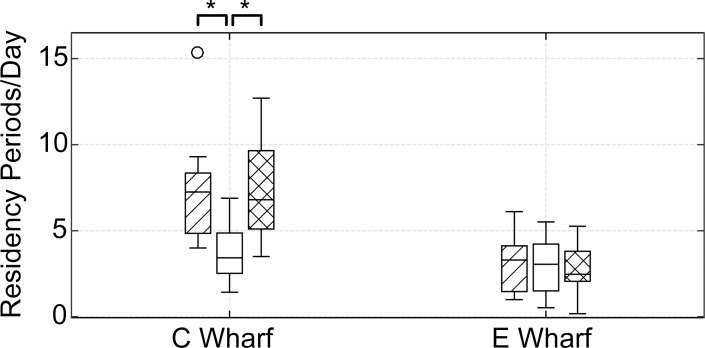
Number of Residency Periods/Day. Residency periods/day are shown for sheepshead during the periods pre- (diagonal lines), during- (open), and post-pile driving (cross-hatched) for the control wharf (C Wharf) and exposure wharf (E Wharf). Asterisks indicate significance at p<0.01.

There were no significant changes in the number of RP/D for sheepshead at the exposure wharf during pile driving for four quarters during pile driving. The median number of RP/D for sheepshead released at the exposure wharf decreased over time from 3.30 (n = 15) to 2.47 (n = 13) pre- to post-pile driving (Fig 6), but no significant differences between time periods were observed. Comparatively, the median number of RP/D for sheepshead released at the control wharf significantly decreased (Mann Whitney U; P<0.01) pre- to during pile driving from 7.27 (n = 12) to 3.43 (n = 12), followed by a significant increase (Mann Whitney U; P<0.01) from during to post pile driving from 3.43 (n = 12) to 6.80 (n = 11).

## Discussion

Based on the results of this study, sheepshead repeatedly exposed to received levels averaging 157 dB re 1 uPa (peak) per pile strike or 162 dB re 1 μPa^2^·s cumulative SEL per pile did not show evidence of displacement from the study area. Of the 13 sheepshead present at the start of pile driving, only one fish left the area after 10 days of pile driving. The remainder were detected on the wharf throughout the duration of pile driving, and 10 sheepshead were still resident on the wharf three months post tagging. Additionally, there were no changes in daytime RI for sheepshead before, during or after pile driving. For grey snapper, only four fish were present at the start of pile driving, and of those two left within the first three days of pile driving. Additionally, there was a non-significant decrease in daytime RI for four snapper with high site fidelity prior to pile driving. While this may constitute a response to pile driving for these fish, the power of statistical tests was limited due to sample size. Both species were monitored in the Canaveral area for several months after pile driving, and results indicate that it is unlikely that the pile driving resulted in mortality or significant injury to tagged individuals.

The peak energy levels based on sound pressure recordings were found to be within the 600–1200 Hz range, a spectrum that was possibly influenced by the attenuation of low frequency sounds in shallower water [20]. It is likely that fish present at the exposure wharf were repeatedly exposed to peak sound pressure received levels in the range of 152 to 157 dB re 1 μPa for each strike, with highest levels in the central portion of the wharf. Fish were not likely repeatedly exposed to peak levels greater than 175 dB re 1 μPa on the outer face of the wharf (and therefore not subject to injury), although grey snapper were periodically observed and caught in this area. Significant attenuation of the pile driving sound along the inside of the wharf, likely due to obstruction from pilings, limited the intensity of sounds in this area creating lower than typical received levels as compared to open water measurements at the same distance, and also limited potential for direct injury or mortality to fish which spent most of their time inside the wharf. The measured ambient 1/3 octave band levels were greater than 100 dB re 1 μPa, which are considered high in comparison with ambient levels presented in other studies [21]. Even though these fish may be accustomed to high background noise levels from regular activity in Port Canaveral, including vessel movement and cargo ship maintenance in Middle Basin, there was still a signal to noise difference of 20–30 dB within the 100–1000 Hz frequency range in the center of the wharf during the pile strikes.

One limitation to the use of these data in developing exposure thresholds for behavioral response is that ground vibrations and particle motion were not directly measured along with sound pressure in the water column. Ground vibration energy from pile driving is typically confined to the seabed [9], and thus had limited potential to directly affect the target species that mostly reside in the upper half of the water column in this environment. Computation of far field distance based on frequency and aperture size [22] showed that receivers E3 and E5 would be considered in the far field where particle motion can be estimated based on a ratio directly related to sound pressure and near-field effects are limited [4]. Receiver E4 at the center of the wharf would also be considered in the far field for frequencies less than 700 Hz. In spite of this information, it would be highly challenging to model or predict what particle motion levels the fishes were actually exposed to in this complicated, shallow acoustic environment based only on the sound pressure data.

Both species of fish in this study possess a swimbladder, although it is unclear if this organ also contributes to detection of sound pressure in these fishes and thus dual sensitivity to both particle motion and sound pressure. Many fishes are able to detect sound pressure in addition to particle motion by use of the gas bladder [4], and the majority of species may possess dual sensitivity [23]. Previous work has detailed swimbladder extensions in the sea bream (*Diplodus annularis*; in the same Sparidae family as sheepshead) that contribute to an increase in hearing sensitivity [24], although it is unknown if sheepshead possess similar anatomical specializations. While the sound field in this environment is very complex and only sound pressure was measured, the relative risk for behavioral impacts to fish resident at the wharf during pile driving was still considered moderate to high with a greatest distance of approximately 225 m from the source [4]. Even if these fish are primarily detecting particle motion, the lack of significant behavioral response at the distances from pile driving observed and the sound pressure levels measured is still relevant in the context of current regulatory analyses and interim sound exposure guidelines.

Sheepshead showed higher site fidelity to the exposure wharf prior to pile driving compared to grey snapper. Species that are dependent on the wharf structure for access to prey, such as sheepshead which mostly forage on sessile invertebrates, may be more at risk of negative consequences from displacement than species that are less dependent on structure as foraging habitat. Therefore, the lack of response shown by sheepshead suggests that there may be limited behavioral response and a low risk of displacement for many structure-dependent fish species from pile driving activities of similar duration and intensity. Additionally, the detection of 10 of the 15 sheepshead present at the start of pile-driving still at the exposure wharf 45 days after pile driving ended suggests that the normal behavior of the fish was not impacted by the 35 day pile-driving event. In fact, six of these sheepshead were still detected in the exposure basin at the end of tag life, almost 1 year after tagging.

Grey snapper are opportunistic predators that likely move more readily between habitats [25], as evidenced by four of the 10 snapper tagged in the exposure basin moving into the control basin prior to the start of pile driving. This species may be more likely to be exposed to higher sound pressure received levels on the outer fringes of the wharf than sheepshead, and more susceptible to displacement. However, given the lower site fidelity evidenced by the majority of grey snapper prior to pile driving, moving to another area may be a more frequent and natural occurrence than for more resident species such as sheepshead, and less of a risk to the fitness of individual fish. Although the three snapper tagged in the control basin showed much higher site fidelity, the control wharf is located in a less open, more protected area, and may reduce movements once fish reach this basin. Sheepshead similarly showed higher site fidelity to the control wharf than the exposure wharf.

Received levels from pile driving experienced by fish in complex coastal environments are dependent on many variables, including the type of pile, method of installation, water depth, and presence of hard bottom or other structure. As a result, sound pressure levels are likely to be highly variable for each installation and difficult to accurately predict. Additional data should be collected to determine the range of possible exposure values from coastal pile driving activities, and measurements of particle motion should be included, particularly in shallow, complex environments. Nonetheless, this study provides a minimum exposure threshold for sound pressure levels within a few hundred meters of pile driving below which no clear behavioral response was observed for fish exposed to pile driving in a natural environment for a duration of several weeks.

Although recent studies have shown strong behavioral responses of fish schools to broadcasted simulated pile driving strikes in a quiet environment [14], important data gaps exist for quantifying sound exposure levels that may result in a substantial behavioral change for a large proportion of the population, particularly for mobile species. Additionally, data are needed for fish with a wide variety of hearing capabilities and morphologies, and should focus on the actual context of the behavioral response and resulting risk. Future studies examining the behavioral response of fish to other impulsive and non-impulsive sources should be conducted in the natural environment of the animals, and attempt to quantify what proportion of the animals exposed exhibit a significant response. Finally, examination of the impacts of high-intensity sound to fish other than reef fish should include potential alteration of migration patterns, site fidelity, distribution, and associated consequences to survivorship.

## Supporting Information

S1 File Extended abstract from: A.N. Popper, A. Hawkins (eds.), *The Effects of Noise on Aquatic Life II*, *Advances in Experimental Medicine and Biology 875*, *DOI 10*.*1007/978-1-4939-2981-8_58*. (PDF)
