# Supplementary material for: Effects of Pile Driving on the Residency and Movement of Tagged Reef Fish
Source: PLoS One. 2016 Nov 23;11(11):e0163638. doi: 10.1371/journal.pone.0163638 (PMC5120787; doi:10.1371/journal.pone.0163638)
Supplement: S1 File — (PDF) [file pone.0163638.s001.pdf]

Chapter 58
Residency of Reef Fish During Pile Driving Within a Shallow Pierside Environment

Joseph D. Iafrate, Stephanie L. Watwood, Eric A. Reyier, Matthew Gilchrest, and Steven E. Crocker

Abstract The potential effects of pile driving on fish populations and commercial fisheries have received significant attention given the prevalence of construction occurring in coastal habitats throughout the world. In this study, we used acoustic telemetry to assess the movement and survival of free-ranging reef fish in Port Canaveral, FL, in response to 35 days of pile driving at an existing wharf complex. The site fidelity and behavior of 15 sheepshead (*Archosargus probatocephalus*) and 10 gray snapper (*Lutjanus griseus*) were determined before, during, and after pile driving. No obvious signs of mortality or injury to tagged fish were evident from the data. There was a significant decline in the residency index for mangrove snapper at the construction wharf after pile driving compared with the baseline, although this may be influenced by natural movements of this species in the study area rather than a direct response to pile driving.

Keywords Anthropogenic noise • Fish • Pile driving • Telemetry • Tagging

J.D. Iafrate (✉) • S.L. Watwood • S.E. Crocker
Environmental Division, Naval Undersea Warfare Center, Newport, RI 02841, USA
e-mail: joseph.iafrate@navy.mil; stephanie.watwood@navy.mil; steven.crocker@navy.mil

E.A. Reyier
Kennedy Space Center Ecological Program, Environmental Services, InoMedic Health Applications, Inc., Kennedy Space Center, Cape Canaveral, FL 32899, USA
e-mail: eric.a.reyier@nasa.gov

M. Gilchrest
Environmental Division, Naval Undersea Warfare Center, Newport, RI 02841, USA

McLaughlin Research Corporation, Middletown, RI 02840, USA
e-mail: matthew.gilchrest.ctr@navy.mil

© Springer Science+Business Media New York 2016

A.N. Popper, A. Hawkins (eds.), *The Effects of Noise on Aquatic Life II*, Advances in Experimental Medicine and Biology 875,
DOI 10.1007/978-1-4939-2981-8_58

lgarcia@reprintsdesk.com

1 Introduction

It has been shown that high-intensity sound sources such as pile driving are capable of inducing injury or hearing threshold shifts in fish at close range to the source (California Department of Transportation 2009; Halvorsen et al. 2011). Although it is expected that an intense source and its associated pressure waves would result in injury to a very small percentage of a population, behavioral changes can occur at greater distances from the source and therefore may affect a larger portion of a population by causing movement of the fish away from a feeding or breeding ground or changes in migratory or communicative behavior (Bridges 1997; Popper 2008; Slabbekoorn et al. 2010).

There is a lack of in-depth behavioral studies examining the effects of high-intensity sounds on fish in the wild (Popper 2008; Slabbekoorn et al. 2010; Normandeau Associates Inc. 2012). The most prominent studies thus far have explored the effects in enclosed environments where behavior cannot be confidently extrapolated to wild animals (Schwarz and Greer 1984; Jørgensen et al. 2005; Popper et al. 2007), the immediate behavioral responses of a single species cannot be investigated (Knudsen et al. 1992, 1994; Gearin et al. 2000), or direct behavioral observations of individual fish could not be included (Culik et al. 2001; Bolle et al. 2012).

In this study, the movement of free-ranging reef fish in Port Canaveral, FL, is documented through the use of acoustic telemetry in response to pile driving. Sheepshead (*Archosargus probatocephalus*) and gray snapper (*Lutjanus griseus*) were chosen as target species due to their abundance, their membership in diverse reef fish families (Sparidae and Lutjanidae), and known high site fidelity to hard-bottom habitats (Reyier et al. 2010). Underwater acoustic receivers were deployed within Port Canaveral to complement an existing array of compatible receivers spanning a range of over 300 km along the east coast of Florida. The study design allowed for a comparison of baseline residency and patterns of movement for unconstrained fish before, during, and after exposure to high-intensity pile-driving sounds.

2 Methods

2.1 Study Area

Port Canaveral is a multiuse harbor that supports cruise ships, fishing ports, and military testing and training activity. Specifically, the Port is composed of a main navigation channel running east–west as well as West, Middle, and Trident (east) Turning Basins (Fig. 58.1). The West Basin contains several cruise ship terminals, while Poseidon and Trident Wharves in the Middle and Trident Basins, respectively, are managed by the Naval Ordnance Test Unit as a US military facility.

The hard-bottom habitat and structure present from the expansive wharves and adjacent stone revetments within the Port create valuable habitats that have resulted

lgarcia@reprintsdesk.com

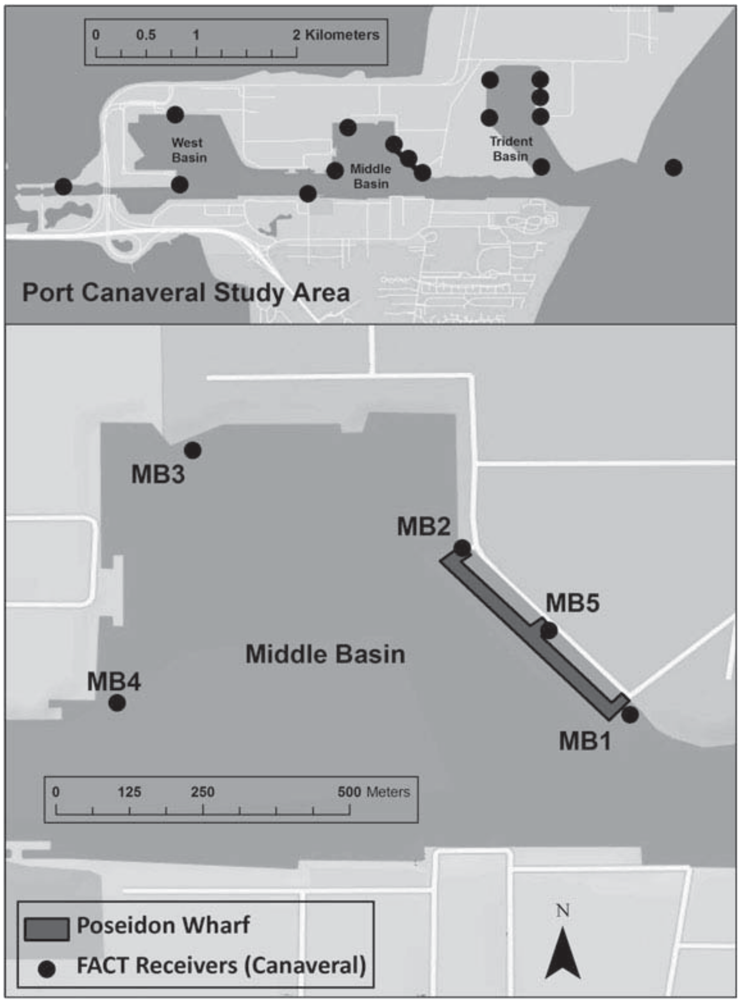

Fig. 58.1 *Top*: Port Canaveral study area with Vemco VR2W receivers (MB1–MB5; *circles*). *Bottom*: Pile driving occurred along Poseidon Wharf in the Middle Basin. *FACT* Florida Atlantic Coast Telemetry array

in robust resident populations of various tropical reef fish species (Reyier et al. 2010). Although these fish are exposed to low levels of anthropogenic noise regularly from activity at Port Canaveral, events that produce noise at the source levels typical of pile-driving construction are rare.

Pile-driving construction was part of a fender replacement project on Poseidon Wharf in the Middle Basin for 35 days in November and December 2011. The project involved removal of expired pressure-treated wooden piles and replacement with 104 polymetric fiberglass-reinforced concrete piles. The square polymetric piles were 16 in. on edge by 80 ft long and placed in two main sections along the

lgarcia@reprintsdesk.com

center and northern outer faces of the wharf. Turbidity curtains were deployed around the pile extraction and placement operations to minimize water turbidity outside the work area. Bottom substrates in the dredged Port Canaveral harbor were considered fine sediment sand or muddy sand, and the approximate depth at the location of the pile driving was 10 m. No construction or other disturbance was occurring in the Trident Basin concurrent with the pile driving along the Poseidon Wharf, and the behavior of tagged fish in that location served as a separate control.

2.2 Acoustic Telemetry

Vemco VR2W autonomous telemetry receivers (Vemco Division, Amirix Systems, Inc.) were deployed in the West, Middle, and Trident Basins to supplement the existing Florida Atlantic Coast Telemetry (FACT) array (Fig. 58.1).

2.3 Collection and Tagging

For this study, a total of 15 sheepshead and 10 gray snapper were tagged in the Middle Basin and 12 sheepshead and 3 gray snapper were tagged in the Trident Basin. All fish were collected by either gill net or hook-line angling. Vemco V9-2 L acoustic transmitters (Vemco Division, Amirix Systems, Inc.) were 29 mm in length and 9 mm in diameter, had a weight of 2.9 g in water (4.7 g in air), and produced a 69-kHz unique coded signal with a nominal burst interval of 60–90 s at 146 dB re 1 µPa at 1 m. Target fish had a minimum weight of 300 g in air to ensure that the tag accounted for no more than 2% of body mass (Winter 1983). Fish were anesthetized in a seawater solution of 75 mg/L of tricaine methanosulfonate (MS-222, Western Chemical, Inc.) and allowed to fully recover in aerated seawater for 5–15 min before release.

Fish were collected 11 days before the start of construction to maximize collection of baseline data. The expected battery life of the Vemco V92L acoustic transmitters was ~11.5 mo.

2.4 Sound Recording

Recordings were made during 4 days of the event at a range of distances from 10 to 370 m from the pile driving. Source levels were measured 10 m from the pile being driven. Ambient recordings were taken when no piles were being driven. Equipment utilized in this effort included two calibrated Cetacean Research Technology (Seattle, WA) C55 hydrophones (mean sensitivity−165 dB re 1 V/µPa), two calibrated High Tech, Inc. (Long Beach, MS) HTI-96-Min hydrophones (mean sensitivity −185 dB re 1 V/µPa), and a DT9837 4-channel dynamic

lgarcia@reprintsdesk.com

signal-acquisition module (Data Translation, Marlboro, MA). Conductivity, temperature, and depth profiles were gathered with a YSI 6920 datasonde (YSI Inc., Yellow Springs, OH).

Acoustic modeling in confined shallow-water environments such as the study site is challenging, particularly in the open spaces of the interior wharf where receivers MB1, MB2, and MB5 were located (Fig. 58.1). As a result, received levels for these areas within the interior portions of the wharf were based on empirical data recorded during the event. For open-water portions of the basins, transmission loss was modeled using the appropriate environmental data (temperature, salinity, and geoacoustic parameters) as defined in the shallow-water propagation-modeling tool (Navy Standard Parabolic Equation [NSPE] Range-dependent Acoustic Model [RAM]), and empirical measurements were utilized as a reference for the transmission loss calculated in the frequency domain. Received levels are presented as root-mean-square (rms) and peak pressure level. The rms values were calculated from the period of individual strikes accounting for 90% of the acoustical energy (California Department of Transportation 2009).

2.5 Residency

Raw detection data were filtered to minimize the probability of accepting false-positive detections (single coded detections within a 30-min window; Pincock 2008). Residency indices (RIs) were calculated for each tagged fish to represent the proportion of days detected on a receiver or group of receivers. RIs were calculated for individual receivers located at Poseidon (MB1, MB2, MB5) and Trident Wharves and for the full combination of receivers located at each wharf to examine broader scale residency at these structures. Baseline comparison of RIs for time periods was conducted utilizing the nonparametric Kruskal–Wallis test and IBM SPSS predictive analytics software. The Wilcoxon matched-pairs signed-rank test was used for post hoc comparisons between paired groups (before to during; during to after; before to after). Significant differences were considered at an α level of 0.017 after a Bonferroni correction for multiple comparisons. Before, during, and after construction timelines were 1–11 November 2011 (days 1–11), 12 November to 16 December 2011 (days 12–47), and 17 December 2011 to 20 January 2012 (days 48–82), respectively. One fish (ID 3023) was caught and harvested by anglers after 75 days of release and was removed from data analysis subsequent to these dates.

3 Results

3.1 Sound Recording and Received Levels

The measured broadband source level using a representative recording of a series of strikes collected 10 m from the pile-driving source was 182 dB re 1 μPa. This recording was utilized as the reference data file for propagation loss as computed by

lgarcia@reprintsdesk.com

NSPE RAM to obtain the received levels in subsequent analyses. Based on measured recordings along the outer and interior wharves, it is likely that fish present within close proximity to Poseidon Wharf were repeatedly exposed to levels in the range of 136–158 dB re 1 μPa rms for each strike over the duration of construction. During pile driving in the central portion of the outer wharf, measured received levels in the vicinity of the telemetry receiver MB5 located in the middle interior portion of the wharf was 136 dB re 1 μPa rms compared with 133 and 139 dB re 1 μPa rms for MB2 and MB1, respectively.

3.2 Residency

The number of fish of both species detected on three of the Middle Basin receivers (MB1, MB2, and MB5) along the interior of Poseidon Wharf from 1 November 2011 through 20 January 2012 is shown in Fig. 58.2. The highest number of fish was detected on receiver MB1, closest to the posttagging release site, followed by MB5, located in the center of Poseidon Wharf. The mean number of unique fish detected per day on the wharf decreased from 15.6 before pile driving to 11.7 and 11.3 during and after pile driving, respectively. No signs of mortality or injury to tagged fish were evident from the data of tagged individuals.

Median RI values for sheepshead in the Middle Basin increased on MB1 (south end of Poseidon Wharf) from 0.3 before construction to 0.5 after construction, while these values decreased on MB5 (closest to the pile driving) from 0.4 to 0.2.

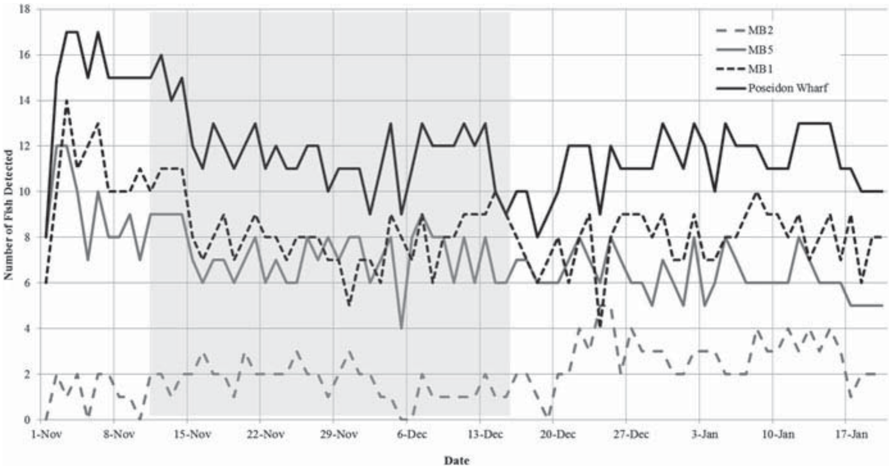

Fig. 58.2 Number of fish detected each day on three Middle Basin receivers (MB2, MB5, and MB1) located within Poseidon Wharf. The postsurgery release site was closest to MB1. Poseidon Wharf represents detection of a particular fish on any of the three receivers. *Gray shading* indicates days of active pile driving

lgarcia@reprintsdesk.com

Residency of sheepshead at MB2 farthest from the tagging release site was consistent with a median of 0 throughout the pile driving, and RI values for the Poseidon Wharf combined receivers stayed relatively constant, ranging from 0.82 before construction to 0.89 after construction. The median values for mangrove snapper decreased on MB1 from 0.45 to 0.01 and from 0.18 to 0.09 on MB5 between the before and after construction periods. The RI values for snapper on MB2 were consistently low, ranging from 0.02 to 0.04, and decreased from 0.58 to 0.18 after construction for the Poseidon Wharf combined receivers. There were significant differences in the RI among time periods for the mangrove snapper on MB1 ($H=7.99$, df $=2$, $P=0.018$) and for Poseidon Wharf combined ($H=9.63$, df $=2$, $P=0.008$), but no significant differences were observed for the sheepshead. Post hoc analysis showed that only the before-to-during construction decrease in the RI for the mangrove snapper on Poseidon Wharf combined was significant ($z=-2.50$, $n=10$, $P=0.013$).

At Trident Wharf, the median RI values for sheepshead ($n=12$) stayed fairly consistent across time periods, with a decrease in values at the receiver located at the middle of the wharf from 0.95 to 0.53 before to after construction. Statistical analysis performed for the sheepshead released in the Trident Basin showed no significant differences among time periods at any of the receivers or for the Trident Wharf combined. Analyses for the mangrove snapper captured in the Trident Basin were not performed due to the small sample size.

4 Discussion

This study was designed to examine the potential changes in residency of unrestrained reef fish in close proximity to impact pile driving. Timing of the fish collection was important to allow sufficient before construction baseline data for comparison with periods during and after construction. Given the success of monitoring tagged, unrestrained fish in the study area for a significant time period, it is unlikely that the pile-driving fender replacement resulted in mortality or significant injury to tagged individuals.

Receivers were deployed to maximize the detection of fish along the interior of Poseidon Wharf and in key areas at the north and south ends. Based on measured recordings along the interior of the wharf and 10 m from the source, it is likely that fish present at Poseidon Wharf were repeatedly exposed to received levels in the range of 136–158 dB re 1 μPa rms for each strike. It is likely that significant attenuation of the pile-driving sound along the inside of the wharf due to obstruction from pilings limited the intensity of sounds in this area and also limited any potential for injury or mortality to fish.

There were no major changes in residency observed for sheepshead along Poseidon Wharf during the pile-driving event; however, there was a significant decrease in snapper residency on Poseidon Wharf receivers from the before to during construction time periods. Mangrove snapper are opportunistic predators

lgarcia@reprintsdesk.com

and likely move between habitats and basins more readily than sheepshead. Poseidon Wharf is also contiguous, with a well-developed subtidal rock revetment, and fish do not have to traverse open water to move away from the wharf or even into the adjacent Trident Basin. As a result, the decrease in snapper residency at Poseidon Wharf during the construction is most likely a result of normal movements for gray snapper in this location.

There are several potential responses of fish to anthropogenic acoustic disturbance, ranging from immediate reactions such as a startle or alarm response to longer term changes in natural behavior. Behavioral response can vary based on a number of factors, including location at the onset of disturbance, dependency on the study area for key life history traits, and habituation to the source over the short term. Mangrove snapper may be more susceptible to displacement because these fish typically school on the fringes of the outer wharf and were therefore potentially exposed to higher levels of sound.

As described above, examination of the potential behavioral impacts to fish species must account for the baseline behavior of fish and characteristics of the study area that may affect individual behavioral response. Additionally, examination of the impacts of high-intensity sound to fish other than reef fish should include potential alteration of migration patterns, site fidelity, distribution, and associated consequences to survivorship.

Acknowledgments We thank the Kennedy Space Center (KSC) Ecological Program; LCDR James Westermeyer and the Naval Ordnance Test Unit; Doug Scheidt and Jane Provancha from InoMedic Health Applications (IHA) at the KSC, FL; and Don George and Angy Chambers at the Air Force 45th Space Wing Natural Assets Office for key logistical support. We also thank Carla Garreau, Russell Lowers, and Karen Holloway-Adkins of IHA; Colin Lazauski, Bert Neales, and Nord Lange of the Naval Undersea Warfare Center (NUWC); and David MacDuffee of US Fleet Forces (USFF). This project was approved by the KSC Institutional Animal Care and Use Committee and the NUWC Environmental Review Board. Funding was provided by USFF Command N46, Canaveral Port Authority, and the NUWC In-House Lab Independent Research Program.

References

Bolle LJ, de John CAF, Bierman SM, van Beek PJG, van Keeken OA, Wessels PW, van Damme CJG, Winter HV, de Haan D, Dekeling RPA (2012) Common sole larvae survive high levels of pile-driving sound in controlled exposure experiments. PLoS ONE 7:e33052

Bridges CM (1997) Tadpole swimming performance and activity affected by acute exposure to sublethal levels of carbaryl. Environ Toxicol Chem 16:1935–1939

California Department of Transportation (2009) Technical guidance for assessment and mitigation of the hydroacoustic effects of pile driving on fish. Final report prepared by ICF Jones & Stokes, Sacramento, CA, and Illingworth and Rodkin, Inc., Petaluma, CA, for the California Department of Transportation

Culik BM, Koschinski S, Tregenza N, Ellis GM (2001) Reactions of harbor porpoises *Phocoena phocoena* and herring *Clupea harengus* to acoustic alarms. Mar Ecol Prog Ser 211:255–260

Gearin PJ, Gosho ME, Lakke JL, Cooke L, Delong RL, Hughes KM (2000) Experimental testing of acoustic alarms (pingers) to reduce bycatch of harbor porpoise, *Phoceoena phocoena*, in the state of Washington. J Cetacean Res Manage 2:1–9

lgarcia@reprintsdesk.com

Halvorsen MB, Casper BM, Woodley CM, Carlson TJ, Popper AN (2011) Predicting and mitigating hydroacoustic impacts on fish from pile installations. National Cooperative Highway Research Program (NCHRP) Research Digest 363, Project 25–28, NCHRP, Transportation Research Board, National Academy of Sciences, Washington, DC

Jørgensen R, Olsen KK, Falk-Petersen IB, Kanapthippilai P (2005) Investigations of potential effects of low frequency sonar signals on survival, development and behaviour of fish larvae and juveniles. Norwegian College of Fishery Science, University of Tromsø, Tromsø, Norway

Knudsen FR, Enger PS, Sand O (1992) Awareness reactions and avoidance responses to sound in juvenile Atlantic salmon, *Salmo salar*. J Fish Biol 40:523–534

Knudsen FR, Enger PS, Sand O (1994) Avoidance responses to low frequency sound in downstream migrating Atlantic salmon smolt, *Salmo salar*. J Fish Biol 45:227–233

Normandeau Associates, Inc. (2012) Effects of noise on fish, fisheries, and invertebrates in the U.S. Atlantic and Arctic from energy industry sound-generating activities. A workshop report prepared under Contract No. M11PC00031 for the Bureau of Ocean Energy Management, US Department of the Interior

Pincock DG (2008) False detections: what they are and how to remove them from detection data. Document No. DOC-004691, version 03, Vemco, Halifax, NS, Canada, 17 April 2012. Available at http://www.vemco.com/pdf/false_detections.pdf

Popper AN (2008) Effects of mid- and high-frequency sonars on fish. Contract N66604-07M-6056, Naval Undersea Warfare Center Division, Newport, RI, Retrieved 21 Feb 2008

Popper AN, Halvorsen MB, Kane E, Miller DL, Smith ME, Song J, Stein P, Wysocki LE (2007) The effects of high-intensity, low-frequency active sonar on rainbow trout. J Acoust Soc Am 122:623–635

Reyier EA, Scheidt DM, Lowers RH, et al. (2010) A characterization of biological resources within the Cape Canaveral Air Force Station Trident Submarine Basin, and adjacent marine waters of Port Canaveral, Florida (May 2008–April 2010). Final report submitted to the US Air Force 45th Space Wing Natural Assets Office, Sept 2010

Schwarz AL, Greer GL (1984) Responses of Pacific herring, *Clupea harengus pallasi*, to some underwater sounds. Can J Fish Aquat Sci 41:1183–1192

Slabbekoorn H, Bouton N, van Opzeeland I, Coers A, ten Cate C, Popper AN (2010) A noisy spring: the impact of globally rising underwater sound levels on fish. Trends Ecol Evol 25:419–427

Winter JD (1983) Underwater biotelemetry. In: Nielson LA, Johnson DL (eds) Fisheries techniques. American Fisheries Society, Bethesda, MD

lgarcia@reprintsdesk.com
